# Comparative Genomic Analysis of Chitinase and Chitinase-Like Genes in the African Malaria Mosquito (*Anopheles gambiae*)

**DOI:** 10.1371/journal.pone.0019899

**Published:** 2011-05-18

**Authors:** Jianzhen Zhang, Xin Zhang, Yasuyuki Arakane, Subbaratnam Muthukrishnan, Karl J. Kramer, Enbo Ma, Kun Yan Zhu

**Affiliations:** 1 Research Institute of Applied Biology, Shanxi University, Taiyuan, Shanxi, People's Republic of China; 2 Department of Entomology, Kansas State University, Manhattan, Kansas, United States of America; 3 Department of Biochemistry, Kansas State University, Manhattan, Kansas, United States of America; 4 Division of Plant Biotechnology, College of Agriculture and Life Science, Chonnam National University, Gwangju, Korea; Ludwig-Maximilians-Universität München, Germany

## Abstract

Chitinase is an important enzyme responsible for chitin metabolism in a wide range of organisms including bacteria, yeasts and other fungi, nematodes and arthropods. However, current knowledge on chitinolytic enzymes, especially their structures, functions and regulation is very limited. In this study we have identified 20 chitinase and chitinase-like genes in the African malaria mosquito, *Anopheles gambiae*, through genome-wide searching and transcript profiling. We assigned these genes into eight different chitinase groupings (groups I–VIII). Domain analysis of their predicted proteins showed that all contained at least one catalytic domain. However, only seven (AgCht4, AgCht5-1, AgCht6, AgCht7, AgCht8, AgCht10 and AgCht23) displayed one or more chitin-binding domains. Analyses of stage- and tissue-specific gene expression revealed that most of these genes were expressed in larval stages. However, *AgCht8* was mainly expressed in the pupal and adult stages. *AgCht2* and *AgCht12* were specifically expressed in the foregut, whereas *AgCht13* was only expressed in the midgut. The high diversity and complexity of *An. gambiae* chitinase and chitinase-like genes suggest their diverse functions during different developmental stages and in different tissues of the insect. A comparative genomic analysis of these genes along with those present in *Drosophila melanogaster*, *Tribolium castaneum* and several other insect species led to a uniform classification and nomenclature of these genes. Our investigation also provided important information for conducting future studies on the functions of chitinase and chitinase-like genes in this important malaria vector and other species of arthropods.

## Introduction

Chitin, a linear polysaccharide of *N*-acetyl-β-D-glucosamine residues joined by β-1,4 glycosidic linkages, is the second most abundant biological polymer after cellulose [1], [2]. It is widely distributed in fungi, nematodes and arthropods. In arthropods, chitin is a vital component of the cuticular exoskeleton and thus is crucial for growth and development [3]. Chitin is also found in internal structures of many insect species and other arthropods, including the cuticular lining of trachea and in the peritrophic matrix (PM) that lines the gut epithelium [4]. During insect growth and development, both the cuticle and PM must be degraded periodically and replaced to allow for growth, maturation and repair. Chitinolytic enzymes play important roles in shedding of the old cuticle and turnover of both the PM and tracheal lining.

Chitinase (EC 3.2.1.14, endochitinase) is an enzyme catalyzing the random hydrolysis of *N*-acetyl-β-D-glucosamine β-1,4 glycosidic linkages in chitin and chitodextrins in a variety of organisms. Chitinases are members of the superfamily of *O*-glycoside hydrolases, which hydrolyze the glycosidic bonds in polysaccharides or between a sugar and a noncarbohydrate moiety. Chitinases have been found in a wide variety of organisms including bacteria, yeasts and other fungi, nematodes, arthropods and even vertebrates such as mice, chicken and human. The vertebrate chitinases probably function as defensive proteins against chitin-containing pathogens. Mammals are not known to synthesize chitin or metabolize chitin as a nutrient; yet the human genome encodes eight GH18 family members that play an important role in T-cell mediated inflammation and asthma [5]–[7].

All insect chitinases belong to family 18 of glycosylhydrolases and many of them may be involved in cuticle turnover, digestion and PM degradation during molting. The first insect chitinase gene cloned was from *Manduca sexta* [8]. In the past, results from cDNA cloning have been interpreted to suggest the presence of a single chitinase gene in each of several insect species including *Chelonas* sp. [9], *Anopheles gambiae* [10], *Bombyx mori* [11], *Spodoptera litura* [12], *Choristoneura fumiferana* [13], *Lutzomyia longipalpis* [14], *Helicoverpa armigera* [15], *Lacanobia oleracea* [16], *Spodoptera frugiperda* [17], *Tenebrio molitor* [18], [19] and *Ostrinia nubilalis* [20]. However, later studies with *B. mori* indicated the presence of multiple chitinase genes [21]. With the completion of several insect genome sequences, rather large and diverse groups of chitinase genes have been identified in many insect species. For example, 16, 16 and 13 chitinase and chitinase-like genes were identified in the genomic databases of the fruit fly, *Drosophila melanogaster*, the red flour beetle, *Tribolium castaneum* and the African malaria mosquito, *An. gambiae*, respectively [22]. Even though the genomes of *Apis mellifera*, *B. mori* and *Aedes aegypti* have not been completely analyzed, available data indicate that chitinases-like proteins are also encoded by multiple genes in these insect species [22], [23].

Based on amino acid sequence similarity and phylogenetic analysis, insect chitinase family proteins have been classified into five groups [22], [24]. Recently, the gene characterization and functional analysis of individual members of the large family of chitinase-like proteins by using gene-specific RNA interference (RNAi) was performed in *T. castaneum*. This research has revealed functional specialization among insect chitinase family genes, primarily during the molting process, and has provided a biological rationale for the presence of a large assortment of chitinase-like proteins [25]. For example, the group I and group II enzymes are involved in molting by digesting cuticular chitin, whereas the group III chitinases have a morphogenetic role in insect development such as regulating abdominal contraction and wing expansion.

*An. gambiae* is an important malaria vector in Africa. To date, only very few chemicals are available for controlling mosquitoes and other human health-related arthropods. Because insect growth and development depend on the precisely tuned chitin synthesis and degradation, chitinolytic enzymes may potentially serve as a selective target for combating insect pests because chitin is not present in vertebrates. In this study we performed a comprehensive genomic analysis of *An. gambiae* chitinase and chitinase-like genes and compared them with those from *D. melanogaster* and *T. castaneum* and several other insect species. Our study is expected to provide a uniform classification of chitinase and chitinase-like genes in insects and to facilitate further research to elucidate the biological functions and physiological significance of the highly diverse chitinase and chitinase-like gene family in insects. A better understanding of biological functions of the individual chitinase and chitinase-like genes may potentially help researchers develop novel strategies for control of arthropod pests by targeting their chitin metabolic pathways.

## Materials and Methods

### Mosquito Rearing

A colony of *An. gambiae* obtained from the Malaria Research and Reference Reagent Resource Center (MR4) (Manassas, VA) was maintained in the Department of Entomology at Kansas State University (Manhattan, KS) since 2007 by using a similar rearing method as described by Zhang and Zhu [26]. Briefly, the larvae were fed with a slurry of brewer's yeast and TetraMin Baby-E fish food, whereas adults were fed with a 10% sucrose solution soaked into cotton balls. Two-day-old females were fed with pre-warmed, defibrinated horse blood (Colorado Serum Company, Denver, CO) in a membrane feeder made of a lubricated Naturalamb brand condom (Church and Dwight Co., Inc., Princeton, NJ) and allowed to lay eggs.

### Genome Search and Sequence Analysis

Five known chitinase and chitinase-like genes from *An. gambiae* were first used as query sequences including a gut-specific chitinase gene (GenBank accession number AAB87764) [10], two partial sequences (GenBank accession numbers AAB81851 and AAB81852) [27] and two bacteria responsive proteins (GenBank accession numbers AAB80137 and AAB80138) [28]. TBLASTN was performed for searching of the *An. gambiae* genome database. Each protein sequence obtained was subsequently used for searching by BLASTp in NCBI. The protein sequences containing the signature sequence FDGXDLDWEYP (highly conserved in all known insect chitinases) and/or one of the other three signature sequences including KXXXXXGGW, MXYDXXG and GXXXWXXDXD were considered as candidate chitinase and chitinase-like proteins [8], [27]. The online program SMART (http://smart.embl-heidelberg.de/) was used to obtain the domain architecture and genomic organization of each gene was conducted by UCSC Genome Bioinformatics program (http://genome.ucsc.edu/). Sequence analysis was performed using the computer software suite Lasergene (DNAstar, WI). The phylogenetic tree was constructed based on domain amino acid sequences using the Neighbor-joining algorithm (Mega 4.0 software). Other software programs utilized from online servers are described in the Results section.

### Reverse Transcription PCR (RT-PCR) Analysis

Total RNA was isolated from mosquito samples representing each of seven developmental stages, including egg, first-, second-, third- and fourth-instar larvae, pupa and adult by using the TRIzol Total RNA Isolation kit (Invitrogen, Carlsbad, CA) for studying stage-specific expressions of *AgCht* genes, 100 eggs, 30 first- or second-instar larvae, 15 third-, fourth-instar larvae, pupae or adults were used for each independent RNA preparation. To study the stage-specific expression in the egg and pupal stages, total RNA was isolated from mosquito samples representing five egg developmental periods collected at 12, 24, 36, 48 and 60 h after oviposition by blood-fed females, and five pupal developmental periods collected at 0, 10, 20, 30 and 34 h after pupation, respectively. Similarly, total RNA was also isolated from tissue samples including the foregut, midgut, hindgut and carcass (whole larva after the gut was removed) for studying tissue-specific expression. In brief, fourth-instar larvae were chilled on ice and dissected in cold 1×PBS to obtain different tissues. The larva was longitudinally opened by carefully cutting the cuticle from one side of the larva without damaging the gut. Then the whole gut was gently removed and detached from adhering tissues including Malpighian tubules, trachea and fatbodies. The midgut, foregut and hindgut were carefully separated and immediately placed in the TRIzol agent. The foregut and midgut were separated at the junction of the gastric caecum and the gastric caecum was included with the midgut. The remaining body tissue excluding the gut was collected as the carcass.

After total RNA was isolated and the concentration determined using the NanoDrop ND-1000 instrument (NanoDrop Technologies, Inc., Wilmington, DE), 2.5 µg of total RNA was then treated with DNase using the DNase I kit (Fermentas, Glen Burnie, MD). First-strand cDNA was synthesized with the First Strand cDNA Synthesis kit (Fermentas, Glen Burnie, MD) using an oligo(dT)_12–18_ primer in a 20-µl reaction volume following the manufacturer's protocol. Beacon Designer software from Primer Biosoft (http://www.premierbiosoft.com) was used to design the gene-specific primers for the genes. The sequences of these primers are shown in supporting information (Table S1). PCR was performed using the PCR Master Mix (Fermentas, Glen Burnie, MD) with a thermal cycle program consisting of an initial denaturation at 94°C for 2 min followed by 29 cycles at 94°C for 30 s, 55°C for 30 s, 72°C for 45 s and a final extension at 72°C for 10 min. The PCR products were resolved on a 1.8% agarose gel and visualized by staining with ethidium bromide. The mosquito ribosomal protein S3 gene (*AgRPS3*) was used as a loading reference for RT-PCR analysis. RT-PCR was repeated at least three times for each gene at each developmental stage and for each tissue. The RNA sample was independently prepared for each of the three replications.

### Real-Time Quantitative PCR Analysis

To confirm the stage- and tissue-specific pression patterns of *AgCht* genes, four of the genes, *AgCht5-1*, *AgCht10*, *AgCht7* and *AgCht8*, were chosen from four different groups (I–IV) for real-time quantitative PCR (qPCR) analysis. cDNA prepared from the above mentioned samples representing each of four developmental stages, including egg, third-instar larva, pupa and adult, and each of four tissues, including foregut, midgut, hindgut and carcass, was used for qPCR analysis. qPCR was performed in a 25-µl reaction volume containing 10.5 µl of 1/10 diluted cDNAs, 0.4 µM of each primer and 1× Maxima SYBR Green qPCR Master Mix (Fermentas, Glen Burnie, MD) using the iCycler iQ real-time PCR detection system (Bio-Rad, Hercules, CA). The optimized qPCR program used for quantification of transcripts for both the *AgRPS3* and targeted *AqCht* genes consisted of an initial denaturation step at 95°C for 5 min followed by 40 cycles at 95°C for 15 sec, 55°C for 30 sec and 70°C for 30 sec. At the end of the PCR, amplification specificity was verified by obtaining the dissociation curve, in which the samples were cooled to 55°C after denaturing and then the melting curves were obtained by increasing 0.5°C/10 s for each cycle with a total of 80 cycles until reaching 95°C to denature the double-stranded DNA. The specificity of each reaction was evaluated based on the melting temperatures of the PCR products. The amplification efficiency of primer pairs was determined from the slope of the curve generated by amplification from serially diluted cDNA. Efficiency had to be at least 0.9 for a primer pair to be accepted. Relative expression values (REVs) for tissue-specific gene expression were then determined by dividing the quantities of the target sequence of interest with the quantity obtained for *AgRPS3* as an internal reference gene.

We found that expression of *AgRPS3* fluctuated across the developmental stages that were tested. Other genes including *AgRPS7*, ribosomal protein L32, elongation factor 2 and the ubiquitin-ribosomal protein L40 fusion protein were also tested. However, none of these was a suitable reference gene to normalize our data across the developmental stages in *An. gambiae* as has been found for other insect species [29]. Therefore, we did not normalize the stage-specific gene expression using *AgRPS3*. Instead, we adopted a similar method [29] by carefully quantifying RNA by NanoDrop measurements to standardize our samples. qPCR was repeated three times for each gene. Each replication was performed based on an independent RNA sample preparation and consisted of two technical replications.

### Immunohistochemical Analysis

Antibody against *M. sexta* chitinase 5 (anti-MsCht5, specific for group I chitinases) and anti-sand fly Cht8 sera, the latter kindly provided by Dr. Ramalho-Ortigao (Department of Entomology, Kansas State University), were used for immunostaining of AgCht5 and AgCht8, respectively, in mosquito pupae. Paraffin-embedded thin sections were used for immunohistochemical analysis. Because the stage-expression pattern showed high expression of both *Agcht5* and *Agcht8* in the pupal stage, pupae were chosen for this analysis. In brief, 12–24 h pupae were fixed in 4% paraformaldehyde at 4°C overnight followed by 3×5 min washes with PBST (PBS and 0.1% Triton X-100). The samples were then dehydrated through an ascending series of ethanol solutions (2×30 min each in 70% and 96%, 2×20 min in 100%), followed by 2×1 h in chloroform. The dehydrated samples were finally embedded in paraffin (56°C, Tyco Healthcare) after overnight penetration. Histological sections (8 µm) were prepared by using a microtome (Richard-Allan Scientific Microm) with a low profile microtome blade (Richard-Allan), straightened on Fisherbrand ColorFrost Plus microscope slides with 0.5% gelatin and allowed to dry for 2 d at 40°C on the top of a slide warmer. The sections were deparaffinized with two washes of 10 min in xylene, rehydrated through successive baths of ethanol (100%, 96% and 70% in water, 1×5 min each), two water washes for 5 min for each and finally PBST for 10 min or more.

For localization of AgCht5 and AgCht8, sections were first blocked using 1% BSA (bovine serum albumin) in PBST for 15 min followed by incubation with a 1∶100 dilution of the anti-MsCht5 or anti-sand fly Cht8 serum in PBST at 4°C overnight. Microsections immunostained with pre-immune serum were used as negative controls. After the sections were washed in PBST three times, each for 2 min, they were incubated with Alexa 488-conjugated goat anti-rabbit (for AgCht5) or anti-mouse (AgCht8) IgG (1∶500 dilution in PBST) at 4°C overnight. After four washes in PBS for 10 min each, the sections were then mounted for 5 min in glycerol containing 300 nM 4′, 6′-diamino-2-phenylindole (DAPI; 2 µg ml^−1^; Sigma) on a glass slide and the fluorescence was observed using a Nikon Eclipse E800 fluorescence compound microscope equipped with appropriate filters. Photographs were taken with a Cool SNAP digital camera.

### qPCR Data Analysis

For qPCR results, relative expression was calculated according to the 2^−ΔΔCt^ method [30]. The data were then transformed using arcsine square root transformation before ANOVA. Fisher's least significant difference (LSD) multiple comparisons were then used to separate the means among the samples.

## Results

### Identification of Chitinase and Chitinase-Like Genes in *An. gambiae*

We identified a total of 20 chitinase and chitinase-like genes in the *An. gambiae* genome based on the presence of signature sequences of insect chitinases by using bioinformatics and transcript profiling approaches (Table 1). This number includes seven new chitinase and chitinase-like genes (*AgCht5-2*, *AgCht5-3*, *AgCht5-4*, *AgCht11*, *AgCht23*, *AgCht24* and *AgIDGF2*) that were identified in this study and 13 others that were previously reported [28]. The GenBank accession numbers of all of these genes are provided in Table 2. The identification of seven new chitinase and chitinase-like genes was mainly due to our recent discovery of a new gene cluster consisting of five duplicated *AgCht5* genes including *AgCht5-1*, *AgCht5-2*, *AgCht5-3*, *AgCht5-4* and *AgCht5-5*. Based on our phylogenetic analysis of the catalytic domains of all of the 20 chitinase and chitinase-like proteins, we renamed the previously reported *AgCht5* and *AgCht11* genes as *AgCht5-1* and *AgCht5-5*, respectively. In addition, we renamed the previously reported *AgBR1* and *AgBR2* genes [28] as *AgIDGF2* and *AgIDGF4*, respectively, to reflect their sequence similarities to other insect imaginal disc growth factors (IDGFs). The IDGFs are chitinase-like proteins that are structurally related to chitinases but do not possess enzymatic activity. They also have an extra loop between the β-4 strand and the α-4 helix of the β8α8 barrel structure of group 18 chitinases [31]. Thus, the total number of *An. gambiae* chitinase and chitinase-like genes is 20, consisting of 2 *AgIDGF* genes and 18 putative chitinase and chitinase-like genes.

**Table 1:** Phylogenetics-based comparative classification of chitinase and chitinase-like genes from three representative insect species.

*D. melanogaster*		*T. castaneum*		*An. gambiae*	
Old	New	Old	New	Old	New
*DmCht1*					
*DmCht2*	*DmCht2*		*TcCht2*	*AgCht2*	*AgCht2*
*DmCht3*					
*DmCht4*	*DmCht4*	*TcCht4*	*TcCht4*	*AgCht4*	*AgCht4*
*DmCht5*	*DmCht5*	*TcCht5*	*TcCht5*	*AgCht5*	*AgCht5-1*
					*AgCht5-2*
					*AgCht5-3*
					*AgCht5-4*
				*AgCht11*	*AgCht5-5*
*DmCht6*	*DmCht6*		*TcCht6*	*AgCht6*	*AgCht6*
*DmCht7*	*DmCht7*	*TcCht7*	*TcCht7*	*AgCht7*	*AgCht7*
*DmCht8*	*DmCht8*	*TcCht8*	*TcCht8*	*AgCht8*	*AgCht8*
*DmCht9*	*DmCht9*	*TcCht9*	*TcCht9*	*AgCht9*	*AgCht9*
*DmCht10*	*DmCht10*	*TcCht10*	*TcCht10*	*AgCht10*	*AgCht10*
*DmCht11*	*DmCht11*		*TcCht11*		*AgCht11*
*DmCht12*	*DmCht12*	*TcCht12*	*TcCht12*	*AgCht12*	*AgCht12*
		*TcCht13*	*TcCht13*	*AgCht13*	*AgCht13*
		*TcCht14*	*TcCht14*		
		*TcCht15*	*TcCht15*		
		*TcCht16*	*TcCht16*	*AgCht16*	*AgCht16*
			*TcCht17*		
			*TcCht18*		
			*TcCht19*		
		*TcCht2*	*TcCht20*		
		*TcCht6*	*TcCht21*		
		*TcCht11*	*TcCht22*		
					*AgCht23*
					*AgCht24*
*DmIDGF1*	*DmIDGF1*				
*DmIDGF2*	*DmIDGF2*	*TcIDGF2*	*TcIDGF2*		*AgIDGF2* b
*DmIDGF3*	*DmIDGF3*				
*DmIDGF4*	*DmIDGF4*	*TcIDGF4*	*TcIDGF4*	*AgIDGF4*	*AgIDGF4* b
*DmCht14*	*DmIDGF5*				
*DmCht13*	*DmIDGF6* a				
18	16	16	22	13	20

a Previously named as *DmDS47* [31].

b *AgIDGF4* and *AgIDGF2* previously named as *AgBR1* and *AgBR2*, respectively [28].

**Table 2:** The accession numbers for revised names of chitinase and chitinase-like genes from three representative insect species.

*D. malenogaster*		*T. castaneum*		*An. gambiae*	
Revised gene name	Accession number	Revised gene name	Accession number	Revised gene name	Accession number
*DmCht2*	NP_477298.2	*TcCht2*	NP_001034516.3	*AgCht2*	XP_315650.4
*DmCht4*	NP_524962.2	*TcCht4*	NP_001073567.1	*AgCht4*	XP_315351.4
*DmCht5*	NP_650314.1	*TcCht5*	NP_001034524.1	*AgCht5-1*	HQ456129
*DmCht6*	NP_572598.1	*TcCht6*	XP_967813.1	*AgCht5-2*	HQ456130
*DmCht7*	NP_647768.2	*TcCht7*	NP_001036035.1	*AgCht5-3*	HQ456131
*DmCht8*	NP_611542.1	*TcCht8*	NP_001038094.1	*AgCht5-4*	HQ456132
*DmCht9*	NP_611543.3	*TcCht9*	NP_001038096.1	*AgCht5-5*	HQ456133
*DmCht10*	EAA46011.1	*TcCht10*	NP_001036067.1	*AgCht6*	*
*DmCht11*	NP_572361.1	*TcCht11*	XP_974461.1	*AgCht7*	XP_308858.4
*DmCht12*	NP_726022.1	*TcCht12*	XP_972802.2	*AgCht8*	XP_316448.2
*DmIDGF1*	NP_477258.1	*TcCht13*	NP_001036034.1	*AgCht9*	XP_307732.4
*DmIDGF2*	NP_477257.2	*TcCht14*	XP_973005.1	*AgCht10*	XP_001238192.2
*DmIDGF3*	NP_723967.1	*TcCht15*	XP_973077.1	*AgCht11*	XP_310662.4
*DmIDGF4*	NP_727374.1	*TcCht16*	NP_001034515.1	*AgCht12*	XP_316142.4
*DmIDGF5*	NP_611321.3	*TcCht17*	XP_972719.1	*AgCht13*	XP_314312.4
*DmIDGF6*	NP_477081.1	*TcCht18*	XP_973161.2	*AgCht16*	XP_319801.4
		*TcCht19*	XP_973119.2	*AgCht23*	XP_001688641.1
		*TcCht20*	XP_970191.2	*AgCht24*	XP_316256.4
		*TcCht21*	NP_001034517.1	*Ag IDGF2*	XP_001237925.1
		*TcCht22*	NP_001038095.1	*Ag IDGF4*	XP_317398.3
		*TcIDGF2*	NP_001038092.1		
		*TcIDGF4*	NP_001038091.1		

*cDNA sequence based on prediction.

**Figure 1:**
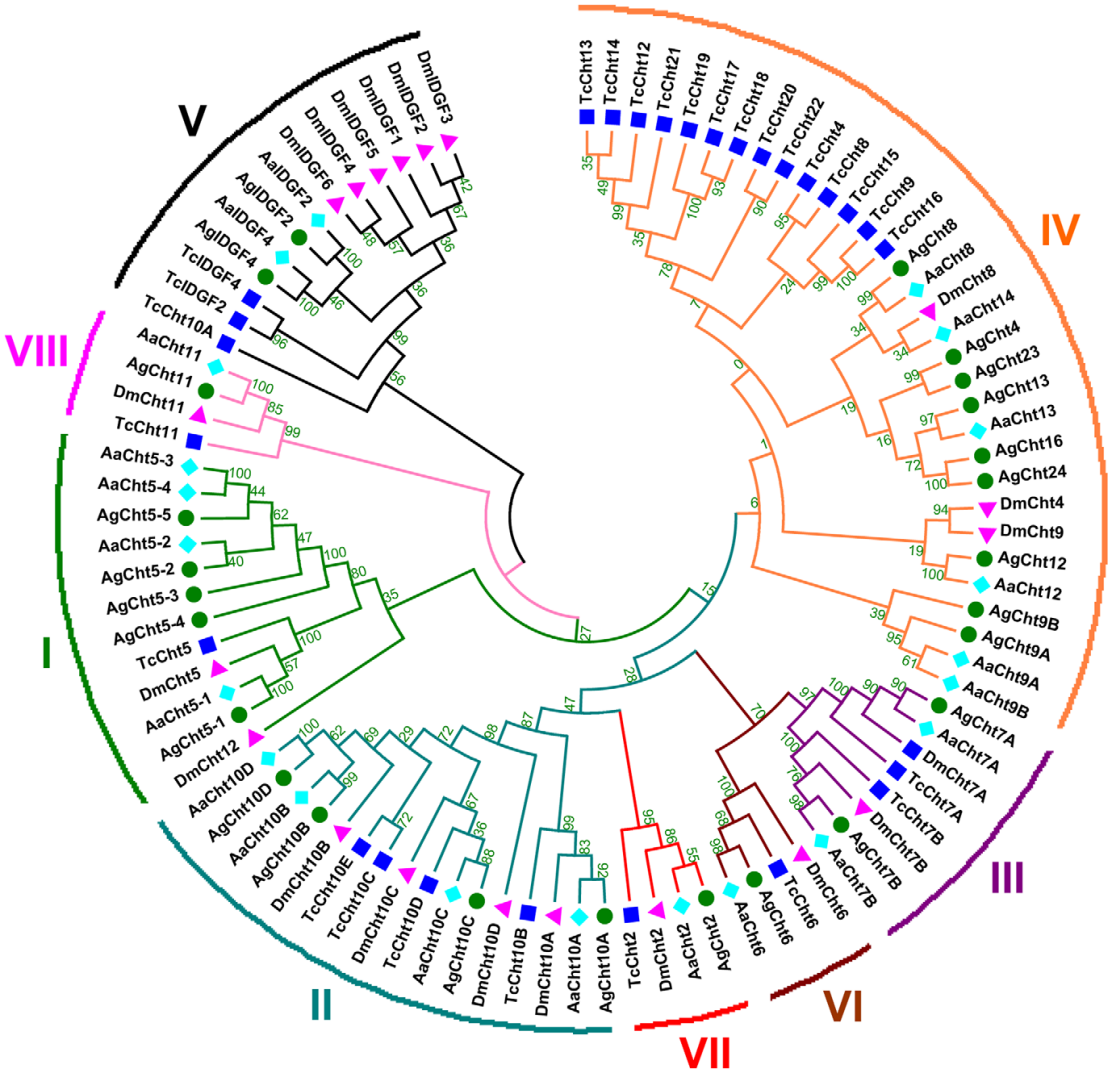
Phylogenetic analysis of chitinase and chitinase-like proteins from three insect species based on catalytic domain sequences. Ag: *An. gambiae*; Aa: *Aedes aegypti*; Tc: *T. castaneum*; Dm: *D. melanogaster*. Phylogenetic tree of insect chitinases generated by the MEGA 4 software after alignment using ClustalW (www.ebi.ac.uk/clustalW). Bootstrap values were obtained by neighbor-joining method using 5000 replications. Protein accession numbers are shown in Table 2 and Table S3.

Phylogenetic analysis based the amino acid sequences of catalytic domains assigned these chitinase and chitinase-like proteins into eight separate groups (I–VIII) (Figure 1). Five groups (I–V) were previously reported in *D. melanogaster* [22] and the remaining three groups, VI, VII and VIII, are closely related but clearly distinct from groups III, II and V, respectively (gene sizes and domain analyses are shown in Table S2). The Cht6 proteins from all three insect species are relatively large proteins that contain 4498, 2369 and 3405 predicted amino acid residues for DmCht6, TcCht6 and AgCht6, respectively.

Six of the eight groups (II, III and VI–VIII) of chitinase and chitinase-like proteins consist of a single chitinase protein in each species, AgCht10 in group II, AgCht7 in group III, AgCht6 in group VI, AgCht2 in group VII and AgCht11 in group VIII, whereas the other three groups, I, IV and V, contain multiple chitinase and chitinase-like proteins that are present in the same insect species. Multiple chitinase 5 proteins belonging to Group I were only identified in the three mosquito species, with five members in *An. gambiae*, four in *Ae. aegypti* and three in *Culex quinquefasciatus*. All of these genes possibly originated from gene duplication events during the evolutionary process. In contrast, chitinase 5 gene duplication was not observed in *T. castaneum*, *D. melanogaster* and any other known insect species. Group IV chitinases are the most divergent and include 3, 14 and 8 chitinase proteins from *D. melanogaster*, *T. castaneum* and *An. gambiae*, respectively. Group V proteins include the putative chitinase-like IDGFs, which are encoded by several genes in each species, for example, 6, 2 and 2 from *D. melanogaster*, *T. castaneum* and *An. gambiae*, respectively. However, three Cht12 proteins from the three insect species are not consistently grouped into the same group. Both TcCht12 and AgCht12 fall into Group IV, the most divergent group of the insect chitinases, whereas DmCht12 falls into Group I.

### Gene Structure of Chitinase and Chitinase-Like Genes

**Figure 2:**
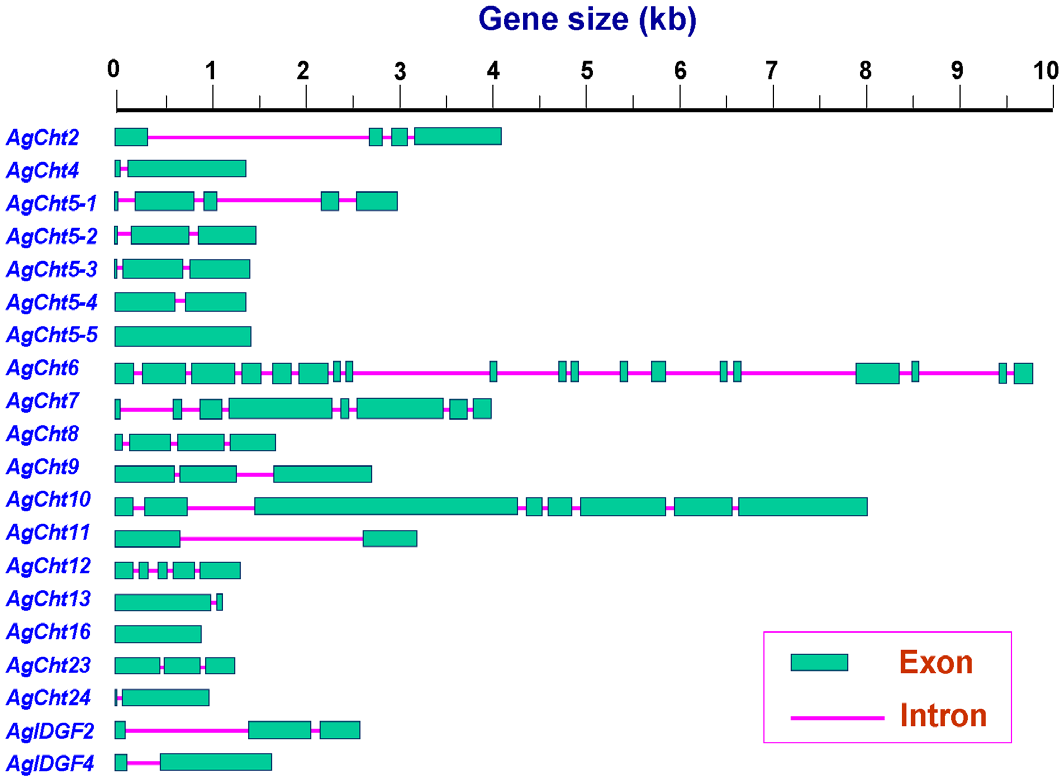
Schematic diagram of the exon and intron organization of the chitinase and chitinase-like genes from *An. gambiae*.

The exon-intron organization of the 20 chitinase and chitinase-like genes is shown in Figure 2. It is clear that the organization of chitinase genes has diverged within the *An. gambiae* genome. A high variation can be observed in both the gene sizes and the number of exons/introns. Some chitinase and chitinase-like genes have as few as only one exon (i.e. without an intron) such as *AgCht16* and *AgCht5-5*, whereas other chitinase and chitinase-like genes have one to several introns. For example, *AgCht6* consists of 19 exons and 18 introns. The sizes of their introns range from less than 100 bp to more than 2 kb.

### Domain Architecture of Chitinase and Chitinase-Like Proteins

**Figure 3:**
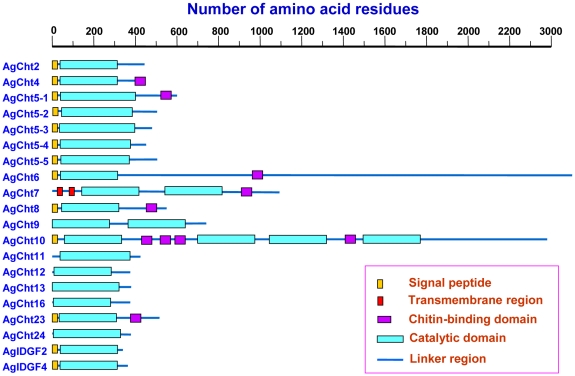
Schematic diagram of the domain architecture of chitinase and chitinase-like proteins from *An. gambiae*.

One of the four conserved motifs in the catalytic domain [2], [3] was used as a signature sequence to identify potential chitinase or chitinase-like proteins for all the three insect species. The consensus sequence, DWEYP, was considered an essential characteristic for a putative chitinase protein. Chitinase and chitinase-like proteins showed extensive similarities at the amino acid sequence level, but a key residue (E) substitution in DWEYP that is known to abrogate catalytic activity is also seen in some of these proteins. Results of the analysis of the domain organization of the deduced chitinase and chitinase-like proteins in *An. gambiae* are shown in Figure 3. Most of them have one catalytic domain except for AgCht7, AgCht9 and AgCht10, which have 2, 2 and 4 catalytic domains, respectively. Seven of 20 chitinase and chitinase-like proteins have one or more chitin-binding domains (CBD) belonging to the ChtBD2 family [31]. Except for AgCht10 with four CBDs, all of the other six chitinase and chitinase-like proteins have only one CBD.

Ten chitinases are predicted to contain a cleavable signal peptide, which suggests that these proteins are secreted proteins that function in an extracellular environment. However, the lack of a signal peptide in the remaining chitinase and chitinase-like proteins in this study could be due to its true absence and/or failure of our predictions when using the SignalP program (http://www.cbs.dtu.dk/services/SignalP/). On the other hand, AgCht7 is the only chitinase that has two predicted transmembrane segments in the N-terminal region. Similarly, at least one transmembrane segment was also found in Cht7 from other insect species including *D. melanogaster*, *A. mellifera* and *T. castaneum* [22], suggesting that Cht7 might be a membrane-anchored protein with the active site facing the outside.

### Expression of *AgCht* Genes in Different Developmental Stages

**Figure 4:**
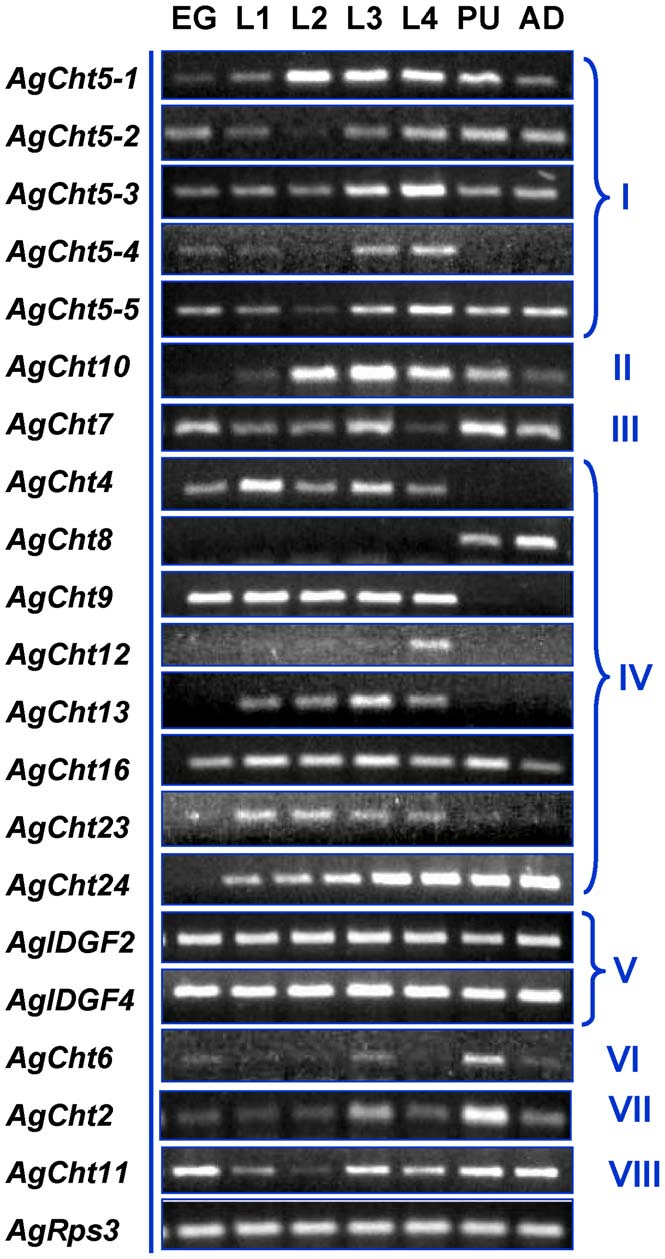
Expression profiling of chitinase and chitinase-like genes in different developmental stages of *An. gambiae* as evaluated by RT-PCR. Eggs (EG,), larvae from first to fourth instars (L1-4), pupae (PU) and adults (AD).

**Figure 5:**
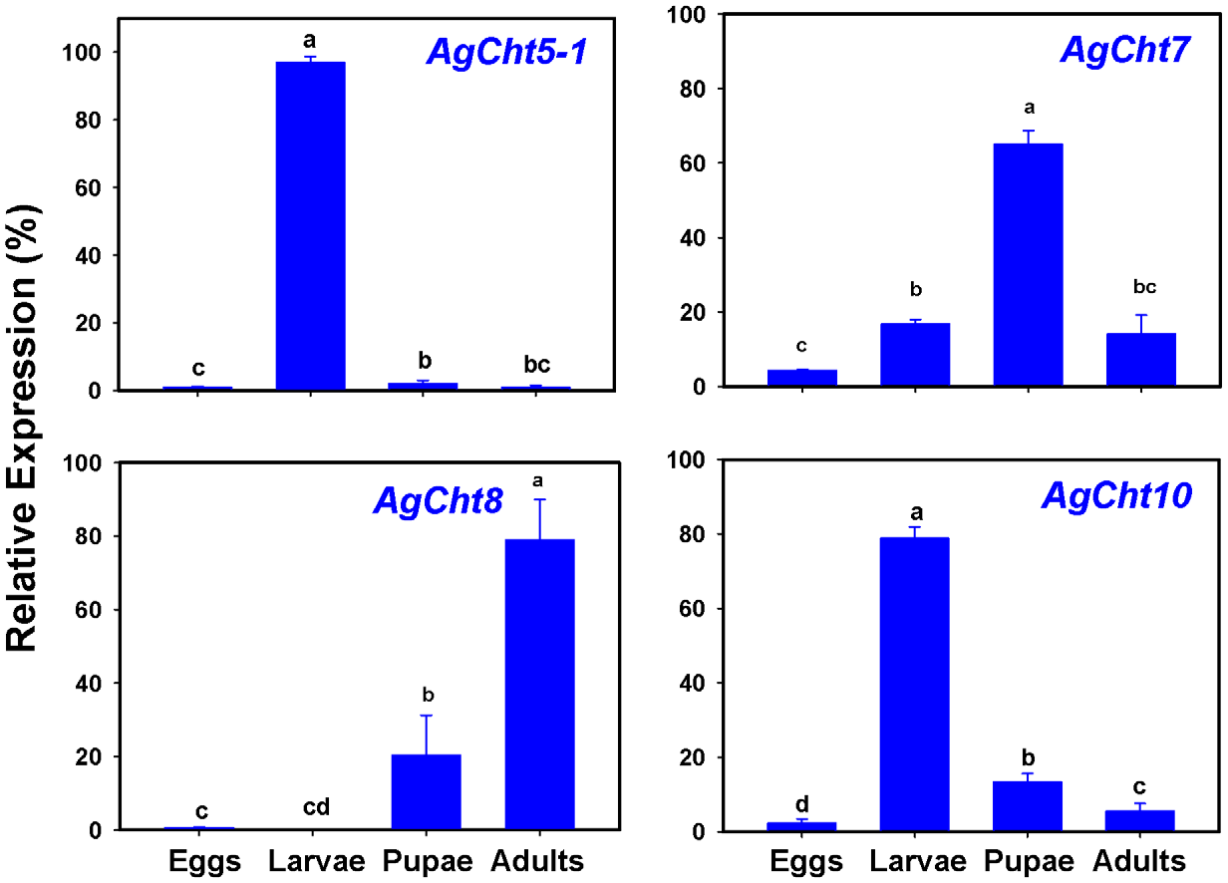
Relative expression of selected *AgCht* genes in different developmental stages of *An. gambiae* as determined by qPCR. Same letters on the error bars indicate no significant difference based on Fisher's LSD test (*P*≥0.05).

Stage-specific expression patterns of *AgCht* genes were determined in embryos (eggs), four different larval instars (first, second, third and fourth), pupae and adults by using RT-PCR (Figure 4). Among the 20 genes, two *IDGF* genes (*AgIDGF2* and *AgIDGF4*) were constitutively expressed in all developmental stages from embryo through adult stages. Ten of the remaining 18 *AgCht* genes, including *AgCht5-1*, *AgCht5-2*, *AgCht5-3*, *AgCht5-5*, *AgCht10*, *AgCht7*, *AgCht16*, *AgCht2*, *AgCht6* and *AgCht11*, showed various levels of expression in all of the seven stages. *AgCht24* was also expressed in most of the stages except for the embryonic stage. In contrast, transcripts for *AgCht5-4*, *AgCht4* and *AgCht9* were detected at various developmental stages from embryo to the fourth-instar larva but not in the pupal and adult stages. Expression of *AgCht8* was detected only in the pupal and adult stages but not in larval stages. Our results also revealed that *AgCht12*, *AgCht13* and *AgCht23* were almost exclusively expressed in the four larval stages, among which *AgCht12* was predominantly expressed in the fourth-instar larva. To confirm our RT-PCR results, the expression of selected genes including *AgCht5-1*, *AgCht7*, *AgCht8* and *AgCht10* were also evaluated by qPCR. The results from qPCR analysis (Figure 5) were consistent with those of the RT-PCR analysis.

We further examined the stage-specific expression patterns of these chitinase and chitinase-like genes in embryos and pupae. RT-PCR analysis was performed in 12-, 24-, 36-, 48- and 60-h old eggs and in 0-, 10-, 20-, 30- and 34-h old pupae. In the eggs, the two IDGF genes were constitutively expressed at all of the periods examined, whereas *AgCht5-2* and *AgCht5-3* appeared to be expressed at all of the times but with some apparent variation in the level of expression (Figure S1). Most of the remaining genes were expressed in the late pupal stages except for *AgCht11*, whose transcripts were detected in the early embryonic stage but gradually decreased thereafter. In pupae most of the chitinase and chitinase-like genes showed various expression levels at all of the selected times of the pupal stage (Figure S2). It was also revealed that *AgCht5-2*, *AgCht5-5* and *AgCht12* appeared to be only expressed in the early pupal stage, whereas *AgCht23* was detected only in the late pupal stage. AgCht13 had a unique expression pattern which was limited to a narrow window in the late pupal stage (30 h).

### Expression of *AgCht* Genes in Different Tissues

**Figure 6:**
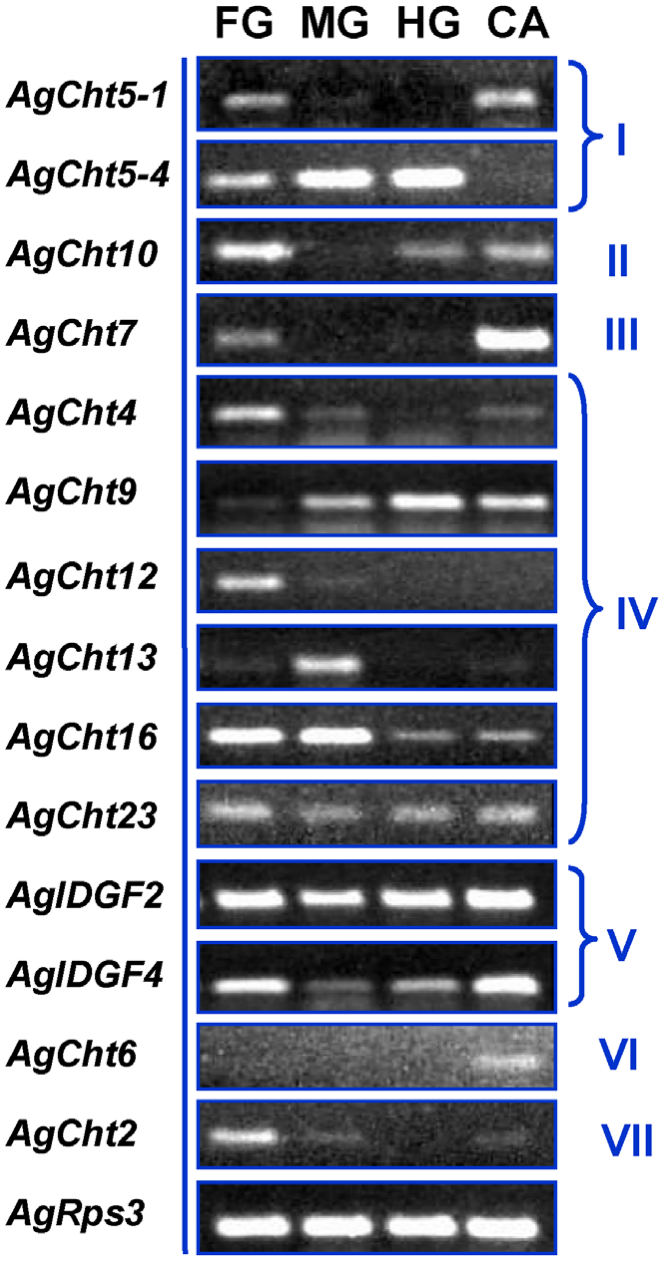
Expression profiling of chitinase and chitinase-like genes in different tissues of *An. gambiae* larvae as evaluated by RT-PCR. Foregut (FG), midgut (MG), hindgut (HG) and carcass (CA).

**Figure 7:**
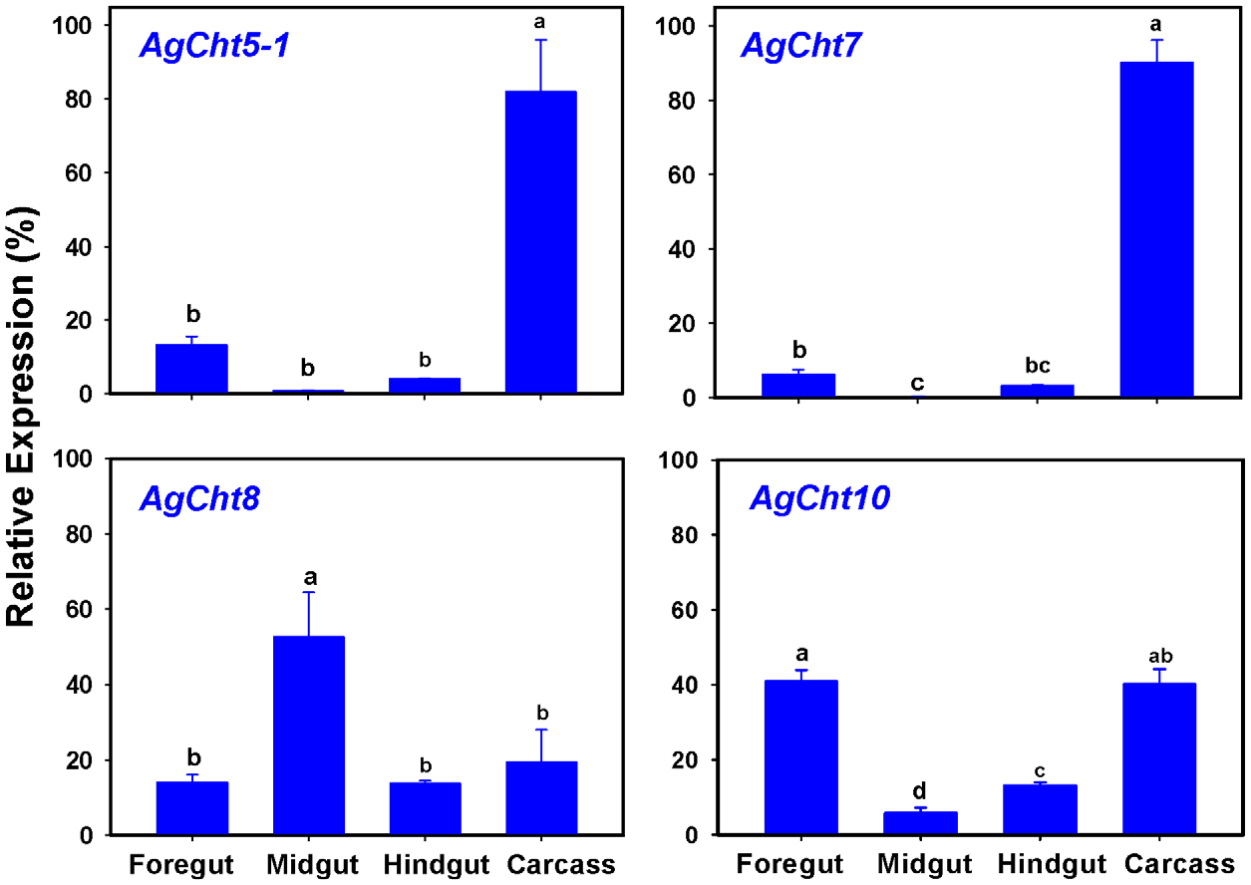
Relative expression of selected *AgCht* genes in different tissues of *An. gambiae* larvae as determined by qPCR. Same letters on the error bars indicate no significant difference based on Fisher's LSD test (*P*≥0.05). The ribosomal S3 (*AgRPS3*) gene was used as a reference gene.

Expression patterns of the 14 *An. gambiae* chitinase and chitinase-like genes were analyzed in each of four selected tissues, including foregut, midgut, hindgut and carcass by using RT-PCR. Our results indicated that six of the 14 chitinase and chitinase-like genes, including *AgCht4*, *AgCht9*, *AgCht16*, *AgCht23*, *AgIDGF2* and *AgIDGF4*, were expressed in all tissues examined, although there apparently were some significant variations in their expression levels (Figure 6). In contrast, *AgCht2* and *AgCht12*, *AgCht13* and *AgCht6* appeared to be exclusively expressed in the foregut, midgut and carcass, respectively. Two *AgCht5* genes showed different expression patterns. *AgCht5-4* appeared to be gut-specific, whereas *AgCht5-1* was expressed predominantly only in the foregut and carcass. In addition, *AgCht7* appeared to be expressed only in the foregut and carcass, whereas *AgCht10* was expressed in the foregut, hindgut and carcass. Further analysis by qPCR revealed that *AgCht5-1* and *AgCht7* were predominantly expressed in the carcass (Figure 7). Expression of *AgCht8* was mainly detected in the midgut, whereas *AgCht10* expression was in the foregut and carcass. The results evaluated by qPCR were consistent with those obtained by RT-PCR. The diverse expression patterns of all of these chitinase and chitinase-like genes may reflect their specialized roles in degradation of chitin in different tissues in mosquitoes as demonstrated in *T. castaneum* [25].

### Localization of AgCht5 and AgCht8 Proteins in Pupae by Immunohistochemistry

**Figure 8:**
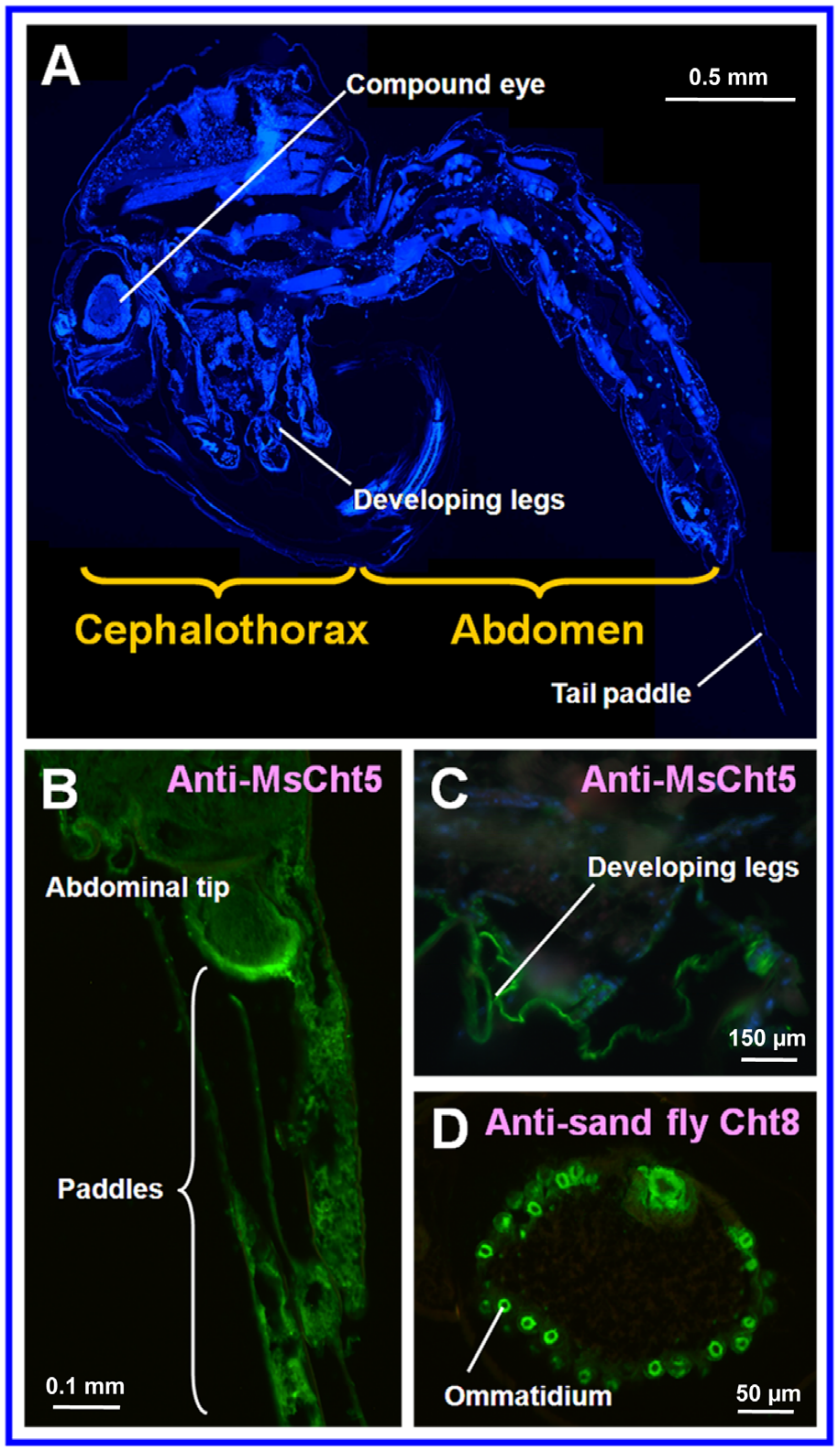
Immunohistochemical localization of selected chitinase proteins expressed in *An. gambiae* pupae. A) A paraffin-embedded thin section of a whole pupa showing the overall structure and corresponding regions where chitinases were detected in immunohistochemical analysis as shown in Panels B, C and D. B) Chitinase detected in the abdominal tip and the tail paddles of a pupa by anti-*Manduca sexta* chitinase 5 polyclonal antibodies (anti-MsCht5) as shown by green color. C) Chitinase detected in certain parts of thorax and developing legs of a pupa by anti-MsCht5 as shown by green color. D) Chitinase detected in the ommatidia of a compound eye by anti-sand fly (*Lutzomyia longipalpis*) chitinase 8 (anti-sand fly Cht8) polyclonal antibodies as shown by green color.

To confirm the diverse expressions of these genes at the protein level in *An. gambiae*, we used readily available anti-*M. sexta* chitinase 5 polyclonal antibodies (anti-MsCht5 for Group I chitinases) and anti-sand fly (*Lutzomyia longipalpis*) chitinase 8 (anti-sand fly Cht8) polyclonal antibodies to localize AgCht5 and AgCht8 proteins, respectively, in paraffin-embedded thin sections of mosquito pupae by using immunohistochemistry. Intensive signals were only observed in certain regions of the head, developing thoracic legs and the abdominal tip including the tail paddles of a pupa when anti-MsCht5 was used in the analysis (Figure 8). However, the high levels of AgCht8 protein were only detected in the pupal compound eyes with an intensive signal in the ommatidia when anti-sand fly Cht8 was used (Figure 8). Because our immunohistochemical analysis showed distinctly different patterns in the localization of AgCht5 and AgCht8, it is unlikely that these antibodies can cross react with AgCht5 and AgCht8. Although we only examined the protein expression for these two genes belonging to two different groups, the results support our hypothesis that different chitinase and chitinase-like genes are expressed in different tissues or body parts where they probably carry out specialized functions.

## Discussion

The availability of whole genome sequences of different insect species has greatly facilitated the identification of chitinase and chitinase-like genes by using a bioinformatics approach. Previous analyses on the chitinase and chitinase-like genes revealed 16 chitinase and chitinase-like genes in *D. melanogaster*, 16 in *T. castaneum* and 13 in *An. gambiae* [22]. Analyses of these genes in *D. melanogaster* and *An. gambiae* were based solely on computational predictions from their genomic sequences. Our extensive search of these insect genomic databases led to the identification of 16, 22 and 20 chitinase and chitinase-like genes in *D. melanogaster*, *T. castaneum* and *An. gambiae*, respectively (Table 1). These new numbers represent the addition of 6 and 7 chitinase and chitinase-like genes to the previous gene numbers reported for *T. castaneum* and *An. gambiae*, respectively [22]. All of the 20 genes from *An. gambiae* were further analyzed by transcript profiling in different developmental stages and in different tissues of the mosquito.

To provide consistent classification and nomenclature of chitinase and chitinase-like genes in different insect species, we re-examined the genome sequences of all three insect species and amended the previous nomenclatures of several genes in these species as shown in Table 1. Compared with previous results from *D. melanogaster* [22], we have determined that the previously assigned two genes previously named *DmCht1* and *DmCht3* were actually only portions of a larger chitinase gene (*DmCht10*) that has four catalytic domains. We re-designated *DmCht14* as *DmIDGF5* and *DmCht13* as *DmIDGF6* based on our phylogenetic analysis of these protein sequences along with those of other insect chitinase and chitinase-like proteins. *DmCht13* was cloned and sequenced in 1995 and previously named as DmDS47 [32]. With all of these changes, we amended the total number of chitinase and chitinase-like genes in *D. melanogaster* to be 16 including 10 putative chitinase and chitinase-like genes and 6 DmIDGF-like genes.

In *T. castaneum*, 16 chitinase and chitinase-like genes were previously identified by isolation and sequencing of chitinase-like cDNAs and BLAST searching of the *T. castaneum* genome database [22]. In this study we revealed 6 new chitinase and chitinase-like genes including *TcCht2*, *TcCht6*, *TcCht11*, *TcCht17*, *TcCht18* and *TcCht19*. In addition, three previously assigned genes, *TcCHT2*, *TcCHT6* and *TcCHT11*, were renamed as *TcCht20*, *TcCht21* and *TcCht22* based on our comparative genomic analysis of the three insect species. Thus, the total number of chitinase and chitinase-like genes in *T. castaneum* was increased from 16 to 22, including 20 putative chitinase genes and 2 *IDGF* genes.

In *An. gambiae*, 13 chitinase and chitinase-like genes were previously identified from its genome database by using bioinformatics approaches [22]. In this study we increased the total number of chitinase and chitinase-like genes to 20. Except for the six genes including *AgCht23*, *AgCht24*, *AgIDGF2* and four *AgCht5* genes (i.e., *AgCht5-2*, *AgCht5-3*, *AgCht5-4* and *AgCht5-5*), each of the 14 remaining genes have putative orthologs in *D. melanogaster* and *T. castaneum* (Table 1). RT-PCR analysis showed that all of the 20 genes were transcribed at some or all of the developmental stages of *An. gambiae* (Figure 4).

We have assembled all of the chitinase and chitinase-like proteins from three insect species into eight groups. If the five *An. gambiae Cht5* genes that apparently arose as a result of gene duplication are not considered, six of eight groups have only a single member, whereas Groups IV and V have multiple proteins in each of the insect species. Group IV, the most divergent group, contains eight chitinase and chitinase-like proteins from *An. gambiae*, six of which are encoded by genes clustered on chromosome 2L, whereas the other two are localized on chromosomes 3L and 3R (Table 3). *AgCht5* appears to be a gene cluster that comprises five different genes that are closely located on chromosome 2R. These genes are likely to be derived from tandem duplications [33]. These results imply that gene duplications and functional divergence resulted in the large number and high diversity of chitinase and chitinase-like genes in different species of insects.

**Table 3:** Predicted numbers of amino acid residues and presence (+) or absence (−) of chitin-binding domains of the proteins putatively encoded by the chitinase and chitinase-like genes, availabilities of expressed sequence tags (ESTs) in GenBank database and the localization of the genes in *A. gambiae* genome.

Gene name	Amino acid residues	Chitin-binding domain	Availability of EST	Chromosomal localization
*AgCht2*	485	−	−	chr2L:17,484,432–17,488,543
*AgCht4*	477	+	+	chr2L:13,688,629–13,690,127
*AgCht5-1*	571	+	+	chr2R:21,584,333–21,587,318
*AgCht5-2*	412	−	+	chr2R:21,582,374–21,583,826
*AgCht5-3*	413	−	+	chr2R:21,578,829–21,580,211
*AgCht5-4*	409	−	+	chr2R:21,576,773–21,578,085
*AgCht5-5*	446	−	+	chr2R:21,573,544–21,574,884
*AgCht6*	3045	+	+	chrX:3,235,497–3,246,126
*AgCht7*	1017	+	+	chr2L:39,004,840–39,009,056
*AgCht8*	525	+	+	chr2L:31,040,019–31,041,796
*AgCht9*	789	−	+	chr3L:13,859,882–13,862,726
*AgCht10*	2402	+	+	chr3R:24,101,945–24,110,279
*AgCht11*	428	−	+	chrX:7713939–7717183
*AgCht12*	382	−	−	chr2L:25,704,430–25,705,882
*AgCht13*	388	−	+	chr2L:4,327,860–4,329,091
*AgCht16*	354	−	+	chr3R:25,026,860–25,027,921
*AgCht23*	442	+	−	chr2L:13,682,087–13,683,505
*AgCht24*	360	−	+	chr2L:27,626,337–27,627,404
*AgIDGF4*	447	−	+	chr3R:4,938,228–4,939,922
*AgIDGF2*	439	−	+	chr3R:4,934,452–4,937,215

The putative proteins encoded by these chitinase and chitinase-like genes were predicted to have a multiple-domain organization that includes 1, 2 or 4 catalytic domains; 0, 1, 4 or 5 chitin-binding domains; 0 or 1 leader signal peptide or transmembrane-spanning domain and linker regions. The domain organizations of the chitinase and chitinase-like proteins in all eight groups from *An. gambiae* showed high similarity to those from *T. castaneum* except for some slight differences [31] as follows. The domain organization of AgCht5-1 from Group I is the same as that of TcCht5. However, *An. gambiae*, *Ae. aegypti* and *C. quinquefasciatus* appear to have 4, 3 and 2 more chitinase 5 proteins as compared to those in *T. castaneum* and *D. melanogaster*. The differences in domain organization of the Group II chitinases (Cht10 s) between *T. castaneum* and mosquitoes have been described in a recent review [31]. The AgCht7 protein in Group III has two N-terminal transmembrane domains, whereas only one transmembrane domain is found in the *T. castaneum* ortholog. In contrast, AgCht11 in Group VIII lacks the N-terminal transmembrane domain that is found in *T. castaneum* Cht11. The most divergent Group IV chitinases of *An. gambiae*, *D. melanogaster* and *T. castaneum* also showed high complexity in their domain organizations. All of the 14 chitinase and chitinase-like *T. castaneum* chitinases in Group IV have a leader signal peptide, whereas in *An. gambiae* only 4 of the 8 chitinase and chitinase-like proteins have a signal peptide. Again, the lack of a signal peptide in the remaining chitinase and chitinase-like proteins could be due to its true absence and/or failure of our predictions by using the SignalP program software. In addition, AgCht9, one member in Group IV, has two catalytic domains but no signal peptide, whereas its counterpart in *T. castaneum* has only one catalytic domain and the signal peptide. Thus, *An. gambiae* has three chitinases with more than one catalytic domain, whereas *T. castaneum* has only two. The phylogenetic analysis and the high similarity of the domain organization of the chitinases belonging to the various groups of chitinases from these two insect species suggest that all of these chitinase proteins evolved from a common ancestor. The preservation of the eight distinct groups with characteristic domain organizations in mosquitoes and the beetle indicates that the appearance of these distinctive groups of chitinases probably predates the separation of the coleopteran and lepidopteran lineages of insects [25], [31].

Stage-dependent expression of these chitinase genes demonstrated substantial differences in expression patterns of individual groups of chitinase and chitinase-like proteins and even between members of the same group with multiple members (Figure 4). The genes encoding chitinase and chitinase-like proteins belonging to Groups I, II, III, V, VI, VII and VIII were expressed in nearly all of the developmental stages from eggs through adult stages with different expression levels, whereas the genes encoding the proteins belonging to Group IV exhibited a high complexity of expression patterns. For example, some genes were only expressed during the larval stages (*AgCht13*), whereas other genes were expressed only in the L4 stage (*AgCht12*) or pupal and adult (*AgCht8*) stages.

The insect chitinase and chitinase-like genes also differed in their tissue-specific expression patterns (Figures 6 and 7). In *T. castaneum*, it appears that all of the Group IV genes are expressed in larval gut tissue, but not in the carcass (whole body minus gut and head) [31]. However, the expression pattern in *An. gambiae* Group IV genes was distinctly different. In *An. gambiae* all of the *Cht* genes of this group were expressed in the foregut including *AgCht9* and *AgCht13*, which were expressed at a lower level than in the midgut. However, it is difficult to separate cleanly the foregut from the midgut by dissection because the mosquito larval foregut is very small and the gastric caecum (GC) belonging to the midgut is often cut off from the midgut and remains with the foregut. Thus, we cannot be sure that the transcripts detected in the foregut truly represent those genes expressed only in the foregut. Similarly, we cannot assign the expression of each chitinase or chitinase-like gene to specific tissues comprising the carcass, which included the fatbodies, trachea, muscle and other tissues.

Nevertheless, one of the most interesting questions about insect chitinases is why insects need such a large number of chitinase and chitinase-like proteins to degrade chitin. In insects, chitin polymorphically occurs in three different crystalline forms, α, β, and γ chitin, that differ in the degree of hydration, in the size of the unit cell and in the number of chitin chains per unit cell [1]. It is possible that insects use different chitinases to efficiently degrade different types of chitin and modified forms such as partially deacetylated chitin. The large number of chitinases expressed in the gut may have digestive and/or immune functions. One supporting piece of evidence for functional diversity among chitinases comes from the fact that there are substantial differences in biochemical properties of chitinase-like proteins belonging to different groups including pH optima and kinetic constants for oligomeric versus polymeric substrates [34], [35].

Furthermore, different forms of chitin could occur in different extracellular structures at different developmental stages. For example, in addition to the chitin in the exoskeleton and peritrophic matrix (PM), chitin and chitin-like material has recently been reported in mosquito eggshells, embryos, ovaries and compound eyes [36]. Different forms of chitin occurring in different extracellular structures may be efficiently degraded by different chitinases. In insects, a compound eye is formed with numerous ommatidia and a part of the ommatidial surface has capacity for secreting chitin because each ommatidium may be regarded as an open pit of the octoderm [37]. As expected, our immunohitochemical anlaysis showed a selective expression of a chtinase protein in the ommatidia of the compound eyes in the mosquito (Figure 8). All these results further suggest the specialized functions of different chitinases in different tissues.

One special characteristic of mosquitoes is that they utilize two types of PMs, a type 1 PM lining the adult midgut that is blood-meal inducible and a type 2 PM lining the larval midgut and constitutively expressed during the whole larval feeding stage. Type 1 and type 2 PMs are different in their thickness and other physiological properties [38]. Our results revealed specialization between two midgut-specific chitinase genes, *AgCht8* and *AgCht13*, as a function of developmental stage. The former is predominately expressed in the pupal and adult stages, but not in the larval stages, whereas the latter is exclusively expressed in the larval stages. These findngs further reinforce the biological significance of the diversity and complexicity of chitinase and chitinase-like genes in mosquitoes. However, additional work is needed to address whether these two chitinases have specialized biochemical properties designed for the turnover of the two types of PMs in the adult versus larval stages of *An. gambiae*.

In summary we have demonstrated that *An. gambiae* chitinase and chitinase-like genes differ significantly in their size, gene structure, domain organization and expression patterns at different developmental stages and in different tissues. All of these results suggest that these genes belonging to different groups or even members within the same group may have distinctly different biological functions. This hypothesis is supported by different physical, chemical and enzymatic properties of different chitinase and chitinase-like proteins from *T. castaneum* and other organisms [34], [35]. This notion is further supported by recent studies showing different phenotypes after different chitinase genes were silenced by RNAi in *T. castaneum* [25]. It appears that Group I and Group II chitinase genes are involved in molting and that Group III genes have a morphogenetic role in regulating abdominal contraction and wing expansion. Some of the members in Group V have been shown to affect cell proliferation in imaginal disks [25]. Although we also performed RNAi for selected chitinase genes in *An. gambiae* by the injection of dsRNA for specific chitinase genes into fourth instar larvae, we did not observe any phenotype due to the lack of or only a limited RNAi response in the larvae (data not shown). Nevertheless, the high diversity and complexity of the chitinase and chitinase-like genes suggest their diverse functions during different developmental stages and in different tissues of *An. gambiae*. Our study has provided important information for further investigations on the functions of chitinase and chitinase-like genes in this important malaria vector and other arthropod species.

## Supporting Information

Figure S1 Expression profiling of chitinase and chitinase-like genes in 12-, 24-, 36-, 48- and 60-h eggs of *An. gambiae* as evaluated by RT-PCR.
(TIF)

Figure S2 Expression profiling of chitinase and chitinase-like genes in 0-, 10-, 20-, 30- and 34-h pupae of *An. gambiae* as evaluated by RT-PCR.
(TIF)

Table S1 Primers used for expression profiling of chitinase and chitinase-like genes in *An. gambiae* by RT-PCR.
(DOC)

Table S2 Comparison of domain architecture of Cht2, Cht6 and Cht11 among *D. malenogaster*, *T. castaneum* and *An. gambiae*.
(DOC)

Table S3 Accession numbers and major protein features of predicted chitinase and chitinase-like genes in *Aedes aegypti*.
(DOC)
