# Supplementary figures and images for: Comparative Genomic Analysis of Chitinase and Chitinase-Like Genes in the African Malaria Mosquito (Anopheles gambiae)
Source: PLoS One. 2011 May 18;6(5):e19899. doi: 10.1371/journal.pone.0019899 (PMC3097210; doi:10.1371/journal.pone.0019899)

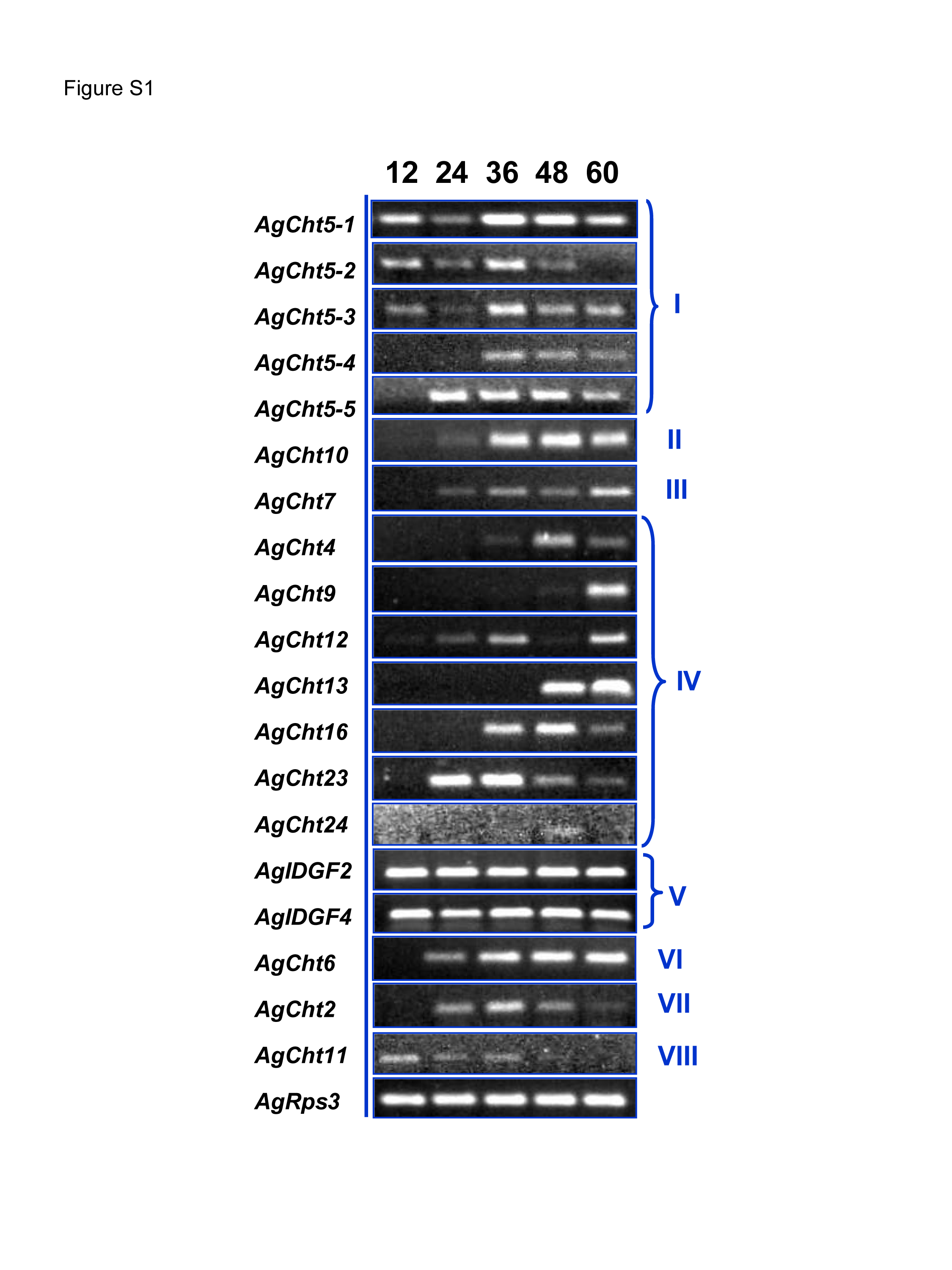

Supplement: Figure S1 — Expression profiling of chitinase and chitinase-like genes in 12-, 24-, 36-, 48- and 60-h eggs of An. gambiae as evaluated by RT-PCR. (TIF) [file pone.0019899.s001.tif]

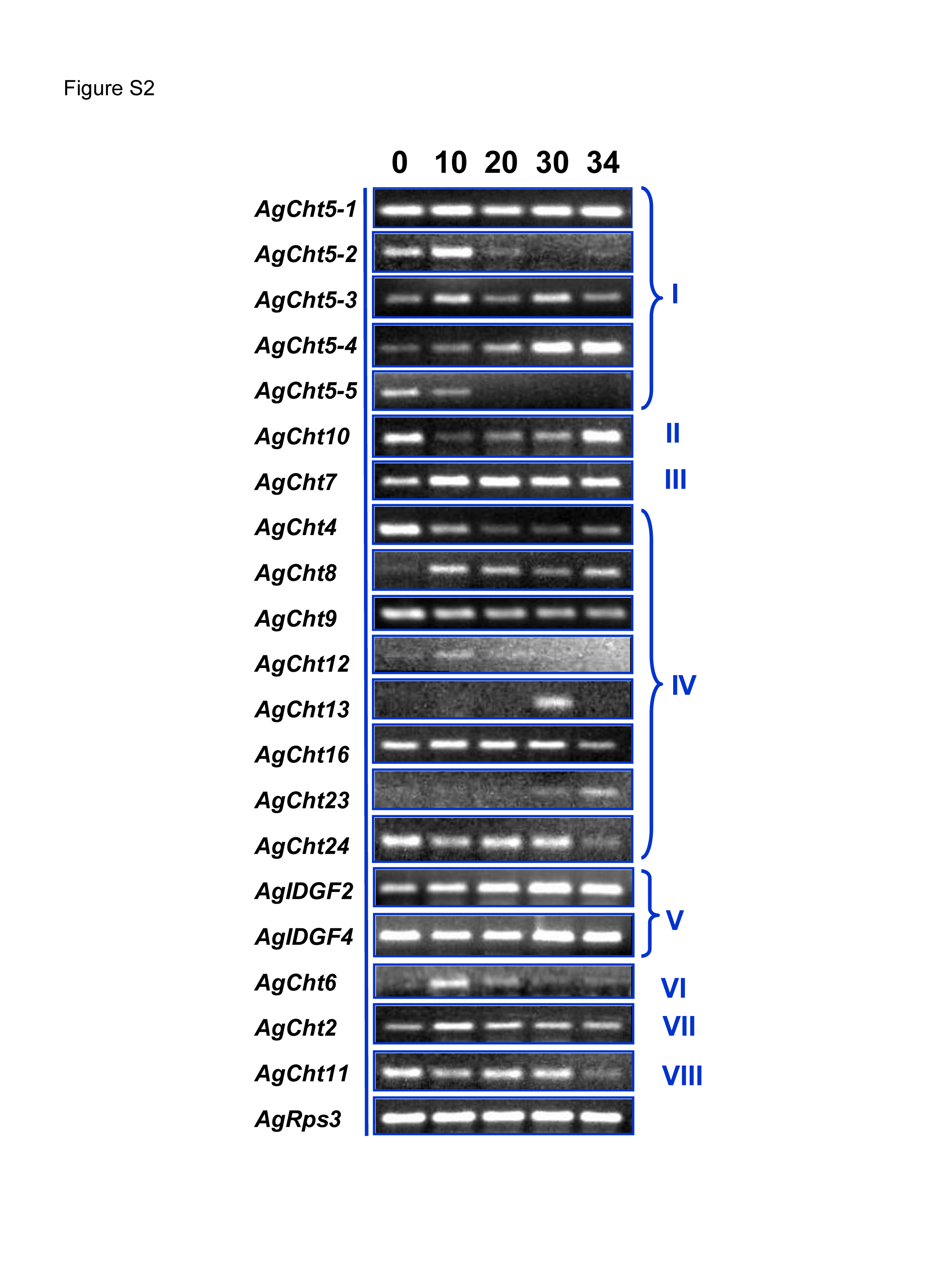

Supplement: Figure S2 — Expression profiling of chitinase and chitinase-like genes in 0-, 10-, 20-, 30- and 34-h pupae of An. gambiae as evaluated by RT-PCR. (TIF) [file pone.0019899.s002.tif]
