# Supplementary material for: Comparative Genomic Analysis of Chitinase and Chitinase-Like Genes in the African Malaria Mosquito (Anopheles gambiae)
Source: PLoS One. 2011 May 18;6(5):e19899. doi: 10.1371/journal.pone.0019899 (PMC3097210; doi:10.1371/journal.pone.0019899)
Supplement: Table S1 — Primers used for expression profiling of chitinase and chitinase-like genes in An. gambiae by RT-PCR. (DOC) [file pone.0019899.s003.doc]

**Supporting Information**

**Table S1.** Primers used for expression profiling of chitinase and chitinase-like genes in *An. gambiae* by RT-PCR.

| Primer Name | Product size (bp) | Primer Sequence (5’-3’) | Tm (^o^C) | GC% |
| --- | --- | --- | --- | --- |
| AgCht2-F | 129 | CTGGATGAGGAGAAGAATGCC | 55.4 | 52.4 |
| AgCht2-R |  | GAGCAGCACCTTCAGATGG | 54.9 | 57.9 |
| AgCht4-F | 137 | AGGGATACTCGCCTACTACG | 54.8 | 55 |
| AgCht4-R |  | TCTGCCCAATACTTTCCACAC | 55.4 | 47.6 |
| AgCht5-1-F | 188 | TTCCGGCTACAAGGACTTTG | 54.8 | 50 |
| AgCht5-1-R |  | TCGGGCTTTCGATCAGTTTC | 55.3 | 50 |
| AgCht5-2-F | 152 | ACGATAAGGACAACTTTGTCTATC | 54.4 | 37.5 |
| AgCht5-2-R |  | GTCAGCACTCTCGCACAG | 54.6 | 61.1 |
| AgCht5-3-F | 166 | GCTGTGTGAAATGCTGAAGG | 54.7 | 50 |
| AgCht5-3-R |  | TGCGTATATGCCACCCAATC | 55.1 | 50 |
| AgCht5-4-F | 146 | TTCGCCAACCTGAAGAAGAC | 55.1 | 50 |
| AgCht5-4-R |  | TGGAGGAACTCAATCACACTG | 54.8 | 47.6 |
| AgCht5-5-F | 197 | TTCATCGGCAGCGTGATC | 54.4 | 55.6 |
| AgCht5-5-R |  | TCGACCGGCACCTGTATC | 55.2 | 61.1 |
| AgCht6-F | 138 | ACTGGTTCAATCTGCTCTCC | 54.2 | 50 |
| AgCht6-R |  | ACTTTACGCTGTAGTCAATGTTG | 55.1 | 39.1 |
| AgCht7-F | 172 | AGTGGCTCAAGGAGGAAGG | 55.2 | 57.9 |
| AgCht7-R |  | GGTCCGAACGACTCATACG | 54.6 | 57.9 |
| AgCht8-F | 141 | TGGAGTGTTAGTGCTAGTTGC | 55.2 | 47.6 |
| AgCht8-R |  | ATGTCATACCGTCCGTTGC | 54.7 | 52.6 |
| AgCht9-F | 139 | ATGGTGTGGTCTATTGAGTCTG | 55.0 | 45.5 |
| AgCht9-R |  | TGGTCTTCGCAGTAGTTGTAG | 54.7 | 47.6 |
| AgCht10-F | 120 | AACAAGGTCCTGTAACATCGG | 55.2 | 47.6 |
| AgCht10-R |  | TACGCTGAGTGGTTGAAGTAG | 54.7 | 47.6 |
| AgCht11-F | 168 | TGGCAACTACATCTACTCGAAG | 55.1 | 45.5 |
| AgCht11-R |  | TTCAGGTGGGTGCAGAGG | 55.1 | 61.1 |
| AgCht12-F | 168 | GTTAAATCGTTGGCTCAAAATGC | 55.6 | 39.1 |
| AgCht12-R |  | ACAATCACCCTGAAAGTCGTC | 55.5 | 47.6 |
| AgCht13-F | 97 | GTCATCTACAACTGGTTGGTATC | 54.4 | 43.5 |
| AgCht13-R |  | TTGCCTATCGTCATATCCTATCC | 54.8 | 43.5 |
| AgCht16-F | 166 | GACCTGAACCCGCACCTG | 56.8 | 66.7 |
| AgCht16-R |  | CACCACCAACCGACACAAG | 55.7 | 57.9 |
| AgCht14-F | 96 | GAACTCCAGGACCAGTCAAG | 54.5 | 55 |
| AgCht14-R |  | GTACCGAATCGTACTGTTTGC | 54.7 | 47.6 |
| AgCht15-F | 150 | TGTTGACTATATCCTCCTGATGAG | 54.9 | 41.7 |
| AgCht15-R |  | CCACCTATCCAATCGCTGAC | 55.1 | 55 |
| AgIDGF2-F | 172 | GTACTCGCTGCTGAAGACG | 55.2 | 57.9 |
| AgIDGF2-R |  | CTCCTCCCGATGCTCCTC | 54.9 | 66.7 |
| AgIDGF4-F | 116 | GTACGACCAGCAGACACC | 53.9 | 61.1 |
| AgIDGF4-R |  | GCCACGCCTTCACCTTATC | 55.3 | 57.9 |
