# Supplementary material for: Comparative Genomic Analysis of Chitinase and Chitinase-Like Genes in the African Malaria Mosquito (Anopheles gambiae)
Source: PLoS One. 2011 May 18;6(5):e19899. doi: 10.1371/journal.pone.0019899 (PMC3097210; doi:10.1371/journal.pone.0019899)
Supplement: Table S2 — Comparison of domain architecture of Cht2, Cht6 and Cht11 among D. malenogaster, T. castaneum and An. gambiae. (DOC) [file pone.0019899.s004.doc]

**Table S2.** Comparisons of domain architecture of Cht2, Cht6 and Cht11 from *D. malenogaster*, *T. castaneum* and *An. gambiae.*

| Gene name | Number of amino acid residues | Number of catalytic domain | Presence or absence of chitin-binding domain |
| --- | --- | --- | --- |
| *DmCht2* | 484 | 1 | - |
| *TcCht2* | 485 | 1 | - |
| *AgCht2* | 485 | 1 | - |
| *DmCht6* | 4498 | 1 | + |
| *TcCht6* | 2369 | 1 | + |
| *AgCht6* | 3045 | 1 | + |
| *DmCht11* | 432 | 1 | - |
| *TcCht11* | 304 | 1 | - |
| *AgCht11* | 428 | 1 | - |
