# Supplementary material for: Comparative Genomic Analysis of Chitinase and Chitinase-Like Genes in the African Malaria Mosquito (Anopheles gambiae)
Source: PLoS One. 2011 May 18;6(5):e19899. doi: 10.1371/journal.pone.0019899 (PMC3097210; doi:10.1371/journal.pone.0019899)
Supplement: Table S3 — Accession numbers and major protein features of predicted chitinase and chitinase-like genes in Aedes aegypti. (DOC) [file pone.0019899.s005.doc]

**Table S3**. The predicted chitinase and chitinase-like accession numbers in NCBI database, amino acid residues, catalytic and CBD domains in *Aedes aegypti.*

| Gene number | Gene name | GenBank No. | Length (aa) | Domain | CBD |
| --- | --- | --- | --- | --- | --- |
| 1 | *AaCht2* | XP_001657537 | 396 | 1 | NS* |
| 2 | *AaCht5-1* | XP_001656234 | 586 | 1 | 1 |
| 3 | *AaCht5-2* | XP_001656233 | 332 | 1 | NS |
| 4 | *AaCht5-3* | XP_001656232 | 413 | 1 | NS |
| 5 | *AaCht5-4* | XP_001656231 | 411 | 1 | NS |
| 6 | *AaCht6* | XP_001662588 | 2816 | 1 | 1 |
| 7 | *AaCht7* | XP_001650020 | 1012 | 2 | 1 |
| 8 | *AaCht8* | XP_001663097 | 469 | 1 | 1 |
| 9 | *AaCht9* | XP_001656054 | 794 | 2 | NS |
| 10 | *AaCht10* | XP_001655973 | 2403 | 4 | 4 |
| 11 | *AaCht11* | XP_001654045 | 513 | 1 | NS |
| 12 | *AaCht12* | XP_001663568 | 391 | 1 | NS |
| 13 | *AaCht13* | XP_001655071 | 382 | 1 | NS |
| 14 | *AaCht14* | XP_001663099 | 501 | 1 | NS |
| 15 | *AaIDGF1* | XP_001660745 | 439 | 1 | NS |
| 16 | *AaIDGF2* | XP_001660748 | 441 | 1 | NS |

* NS: Not shown in prediction.
